# Synthesis and Deposition of Silver Nanowires on Porous Silicon as an Ultraviolet Light Photodetector

**DOI:** 10.3390/nano13020353

**Published:** 2023-01-15

**Authors:** Anas A. M. Alqanoo, Naser M. Ahmed, Md. R. Hashim, Munirah A. Almessiere, Sofyan A. Taya, Ahmed Alsadig, Osamah A. Aldaghri, Khalid Hassan Ibnaouf

**Affiliations:** 1 School of Physics, Universiti Sains Malaysia, Gelugor 11800, Penang, Malaysia; 2 Physics Department, Islamic University of Gaza, Gaza P.O. Box 108, Palestine; 3 Research Center, The University of Mashreq, Baghdad 10021, Iraq; 4 Department of Physics, College of Science, Imam Abdulrahman Bin Faisal University, P.O. Box 1982, Dammam 31441, Saudi Arabia; 5 Department of Biophysics, Institute for Research and Medical Consultations (IRMC), Imam Abdulrahman Bin Faisal University, P.O. Box 1982, Dammam 31441, Saudi Arabia; 6 CNR NANOTEC Institute of Nanotechnology, Via Monteroni, 73100 Lecce, Italy; 7 Physics Department, College of Science, Imam Mohammad Ibn Saud Islamic University (IMSIU), Riyadh 13318, Saudi Arabia

**Keywords:** AgNWs, sheet resistance, photodetector, absorption, porous silicon

## Abstract

The applications of silver nanowires (AgNWs) are clearly relevant to their purity and morphology. Therefore, the synthesis parameters should be precisely adjusted in order to obtain AgNWs with a high aspect ratio. Consequently, controlling the reaction time versus the reaction temperature of the AgNWs is crucial to synthesize AgNWs with a high crystallinity and is important in fabricating optoelectronic devices. In this work, we tracked the morphological alterations of AgNWs during the growth process in order to determine the optimal reaction time and temperature. Thus, here, the UV–Vis absorption spectra were used to investigate how the reaction time varies with the temperature. The reaction was conducted at five different temperatures, 140–180 °C. As a result, an equation was developed to describe the relationship between them and to calculate the reaction time at any given reaction temperature. It was observed that the average diameter of the NWs was temperature-dependent and had a minimum value of 23 nm at a reaction temperature of 150 °C. A significant purification technique was conducted for the final product at a reaction temperature of 150 °C with two different speeds in the centrifuge to remove the heavy and light by-products. Based on these qualities, a AgNWs-based porous Si (AgNWs/P-Si) device was fabricated, and current-time pulsing was achieved using an ultra-violet (UV) irradiation of a 375 nm wavelength at four bias voltages of 1 V, 2 V, 3 V, and 4 V. We obtained a high level of sensitivity and detectivity with the values of 2247.49% and 2.89 × 10^12^ Jones, respectively. The photocurrent increased from the μA range in the P-Si to the mA range in the AgNWs/P-Si photodetector due to the featured surface plasmon resonance of the AgNWs compared to the other metals.

## 1. Introduction

The ever-growing demand of the functional photoelectric devices and scarcity of indium has posed new challenges to researchers worldwide in developing novel flexible transparent conductive films (TCFs) that are the essential components of optoelectronic devices. It has been realized that traditional indium tin oxide (ITO) can be replaced by alternative candidates like carbon nanotubes (CNTs), graphene, metal grids, conductive polymers, and silver nanowires (AgNWs) [1,2,3,4,5,6,7]. Among these substitutes, silver nanowires have a superior electrical conductivity and transmittance to carbon-based materials [8]. Meanwhile, several studies indicated that metal grids and AgNWs may be the best options to substitute ITO [9]. However, metal grid manufacturing is costly, and causes a substantial moiré interference that is detrimental to the devise’s performance [10,11]. Compared to the metal grids, the AgNWs are highly transparent to visible light, electrically conducting, as well as mechanically flexible [12]. Over the years, several techniques have been developed to produce high-quality AgNWs with tailored traits. Commonly, the simple polyol method is used to produce AgNWs with varying shapes and sizes by dissolving AgNO_3_ in C_2_H_6_O_2_ and metal halide (C_6_H_9_NO)_n_ that acts as a stabilizer [13,14,15]. A wide range of conditions is believed to influence the nucleation and growth mechanisms of the AgNWs, including the molecular weight of (C_6_H_9_NO)_n_, the growth time and temperature, the injection rate of AgNO_3_, the molar ratio of (C_6_H_9_NO)_n_ to AgNO_3_, the stirring speed, and the halide anions concentration [16,17,18,19,20,21]. Jiu et al. used a basic polyol method and varied the stirring speeds to grow very long (80 µm) AgNWs with a diameter of 80 nm [15]. In another work, Atwa et al. used a high temperature (170 °C) to produce AgNWs with diameters above 35 nm [22]. Kim et al. showed that AgNWs with a diameter of 62.5 nm and a length of 13.5 µm can be produced using 4 h of growth duration [23]. Despite many efforts, the actual growth mechanisms of the AgNWs as well as a correlation between the growth temperature and time have still not been fully explored.

A wide range of applications, such as optoelectronic, biosensors, communications, and photonics, depend on photodetectors, which transform the optical energy into photoelectrons. The development of high-quality photodetectors is attributable to the scientific work on 1-D nanostructures. Even though 1-D nanostructured material photodetectors have received an extensive design and performance improvement, work on developing photodetectors is still ongoing, and researchers are making unremitting efforts for this purpose [24]. Many AgNW films for photodetector applications have been fabricated [25,26,27,28]. These AgNW hybrids have demonstrated a variety of intriguing phenomena, such as a distinctive charge separation and their surface plasmonic properties.

In this work, we prepared AgNWs using the standard polyol method at a varying growth time with the temperature. Our central idea was to demonstrate the impact on the AgNWs’ production quality as a function of both the temperature and reaction time. In addition, we empirically developed an equation regarding the growth rate at various temperatures based on the obtained optical spectroscopic profiles. We also highlighted a unique purification approach to purify the AgNWs. Finally, the purified AgNWs were used to enhance the responsivity of the P-Si photodetector to UV light. The AgNWs/P-Si photodetector has been investigated under four bias voltages using UV light of a 375 nm wavelength.

## 2. Materials and Methods

### 2.1. Materials

Pure chemical reagents of polyvinylpyrrolidone (PVP 1300 k), silver nitrates (AgNO_3_), sodium chloride (NaCl), and potassium bromide (KBr) were purchased from Sigma Aldrich company (St. Louis, MO, USA). Ethanol (99.5%), acetone, and ethylene glycol (EG) were purchased from a chemical store in Universiti Sains Malaysia (USM). Deionized water (DIW) was obtained in the NOR lab in the school of physics (USM).

### 2.2. Preparation of AgNWs by Polyol Method

First, NaCl (0.25 M), KBr (0.25 M), and 6 mL of PVP (0.5 M) solutions were prepared. Then, 13 mL of EG were taken in a flask of a 50 mL volume before being immersed in a preheated oil bath for 20 min at 150 °C and stirred vigorously at a speed of 800 rpm. Later, the prepared solutions of NaCl, KBr, and PVP were inserted in the flask and kept for 7 min. Finally, AgNO_3_ (0.15 g) was dissolved with EG (7 mL) and then added gradually at 0.25 mL/min for the duration of 28 min and allowed to react continuously until a greenish-grey colour solution appeared. During the process, small amounts of the solution were repeatedly taken at different times and the absorption spectra were recorded at each time in order to obtain the sharpest absorbance and estimate the reaction time for each temperature. The arrival of a growth time indicated the end of the reaction for the AgNWs’ formation. The resultant mixture containing AgNWs was cooled down to an ambient temperature and purified for further characterizations.

### 2.3. Preparation of Porous Silicon Substrate

The P-Si was fabricated using the electrochemical etching technique. In the fabrication process, the optimal parameters were applied [29]. First, a 400 μm thick, n-type Si peace with an area of 1 cm^2^ and resistivities between 1 and 10 Ω.cm. An electrochemical cell made of Teflon was then filled with a 1:7 volume ratio combination of ethyl alcohol and hydrofluoric acid (HF). The Si peace was then positioned into the bottom of the cell Teflon and fastened with a copper board as an anode or cathode. As the cathode, a tungsten wire was introduced at a distance of 1 cm from the Si surface. The cell was illuminated by a tungsten lamp as a light source during the etching process, with an etching time of 30 min and a 0.04 A/cm^2^ current density. A brown disc-shaped P-Si was produced after the etching procedure. When subjected to UV light, the surface of the P-Si disc that is generated produces an orange-red shine, demonstrating that the P-Si was successfully produced via electro-chemical etching.

### 2.4. Characterization of the Prepared Samples

The UV–Vis–NIR absorbance and morphology of the samples at various temperatures and times were recorded using a Cary 5000 (Agilent, 5301 Stevens Creek Blvd, Santa Clara, CA 95051, USA) absorption spectrophotometer and an FEI Nova SEM 450 field emission scanning electron microscope (FESEM, Hillsboro, OR, USA), respectively. The presence of AgNWs in the solution and their morphology were analyzed by a Libra 120 transmission electron microscope (TEM, Zeiss GmbH, Germany). Atomic force microscopy (AFM) was conducted with a Bruker Multi-Mode 8 scanning microscope.

## 3. Results and Discussion

### 3.1. The Growth Mechanism of AgNWs

**Figure 1:**
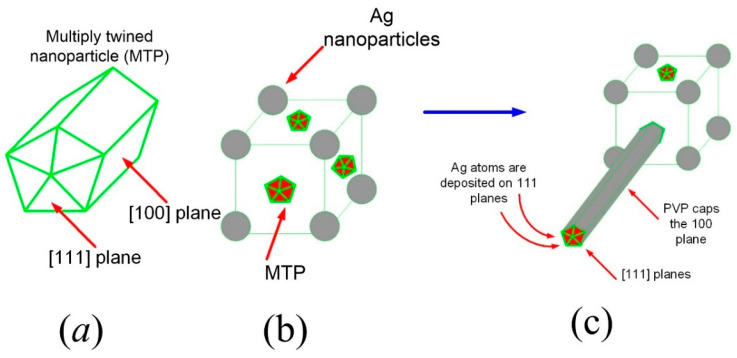
Schematic diagram for the growth mechanism of (**a**) MTPs, (**b**) AgNPs, and MTPs-decorated nanocrystals, and (**c**) anisotropically elongated AgNWs from the nanocrystal surface.

Figure 1a–c shows the schematic diagram for the growth mechanism of the AgNWs with the formation of multiple twinned nanoparticles (MTPs) and silver nanoparticles (AgNPs). The MTPs decorated nanocrystals and anisotropically elongated Ag nanorods (AgNRs) from the surface of the nanocrystals. In this work, the NaCl to KBr molar ratio (which acted as a control agent) was kept at 2:1 to create insoluble AgCl, AgBr, and silver halide (AgBr_1-x_ Cl_x_) crystals decorated with silver seeds in the early reaction phases. After a short period, some of these seeds developed into MTPs (Figure 1b). Each MTP consisted of ten facets, with five [111] facets on each end and other five [100] facets on the lateral surface (Figure 1a). Furthermore, some of these MTPs remained entirely within the crystal and others resided on the crystal’s surface. The diameters of the produced AgNWs were strongly depended on the size of the MTPs, that in turn depended on the crystal’s components, indicating their higher dependence on AgCl than on AgBr. Consequently, the lengths and diameters of the AgNWs prepared using Cl salts were larger than the ones produced by Br salts. This issue was resolved by using two halides (Cl and Br) at a fixed ratio, thus obtaining AgBr_x_ Cl_x-1_ crystals with the desirable sizes of the MTPs and the eventual growth of the AgNWs with a high aspect ratio.

### 3.2. Spectroscopic Analysis

**Figure 2:**
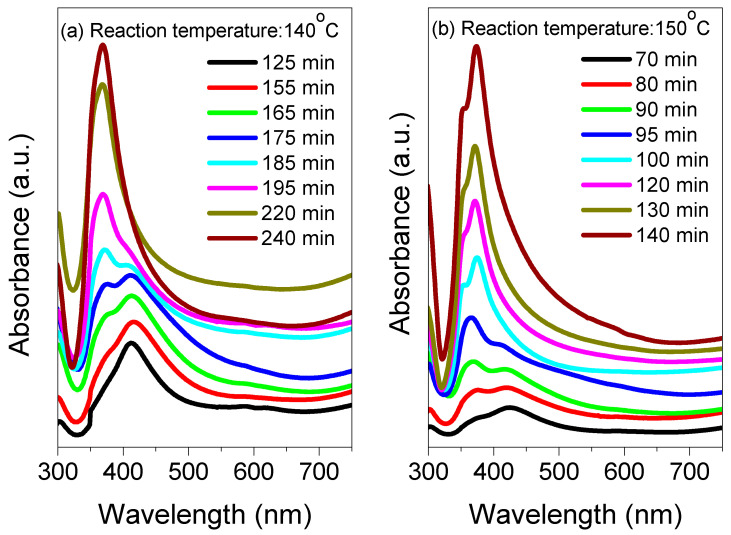
The absorbance of AgNWs suspension for the growth duration in the range of (**a**) 125–240 min at a reaction temperature of 140 °C, and (**b**) 70–140 min at a reaction temperature of 150 °C.

**Figure 3:**
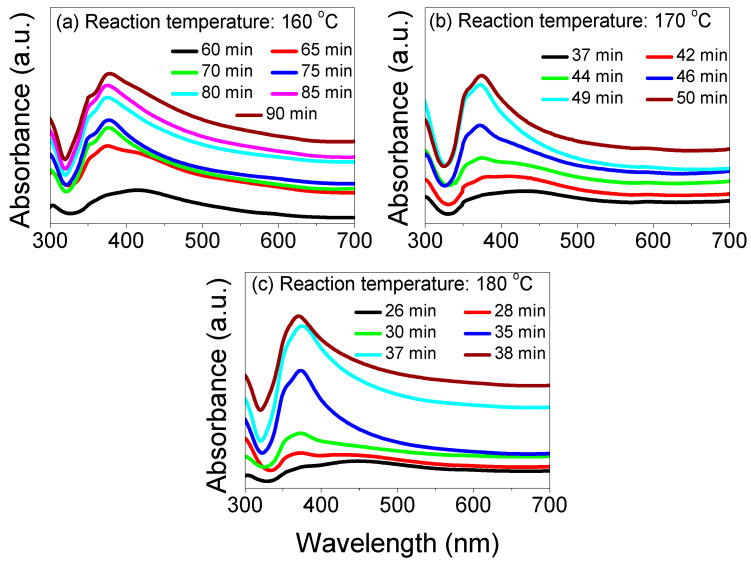
The absorbance of AgNWs suspension for the growth duration in the range of (**a**) 60–90 min at a reaction temperature of 160 °C, (**b**) 37–50 min at a reaction temperature of 170 °C, and (**c**) 26–38 min at a reaction temperature of 180 °C.

Figure 2 and Figure 3 illustrate the reaction temperatures (140, 150, 160, 170, and 180 °C)-dependent UV–Vis–NIR absorption spectra of the AgNWs recorded at various times (26 to 240 min), indicating their strong influence on the growth evolution of AgNWs. The absorption spectra were further analyzed to determine the conversion process of AgNPs into AgNWs, in addition to an estimate of the reaction time that occurs when the spectrum peak becomes the sharpest. Figure 2a shows that at a reaction time of 125 min, only AgNPs were formed at 140 °C. The absorption spectrum is symmetric with a plasmon resonance peak at 415 nm. At 155 min, the spectrum at wavelengths less than 400 nm showed a convex shape, suggesting the formation of silver nanorods (AgNRs) from the Ag nanocrystal’s surface. At a growth time of 165 min, a shoulder was observed at around 380 nm, which was due to the elongation of the AgNRs. This shoulder showed a red-shift and became more prominent with the further increase in the growth time at 175. This clearly indicated an increase in the AgNRs density at the expense of AgNPs. At 185 min, the shoulder transformed into a peak of about 376 nm. In addition, the absorption peak was blue-shifted to 370 nm at 195 min, respectively (Figure 2a). The absorption spectra above 400 nm remained broad, confirming the incompleteness of the AgNWs’ growth and that AgNPs are still existing in the solution. At 220 min, the peak due to AgNPs disappeared, leaving behind the AgNWs’ peak. It is important to determine the completion time (called the growth time) of the AgNWs’ growth. The state-of-the-art growth methods of the AgNWs in the literature seldom address the exact growth time at different temperatures that generally occur when the absorption peak becomes the sharpest and the growth solution is devoid of AgNPs. Conversely, a broad band appears without any sharp peak if the reaction is incomplete, implying the degradation of AgNWs. In the present case, the occurrence of the sharpest peak at a growth temperature of 140 °C clearly indicated a growth time of 220 min. After an additional 20 min, the spectrum broadened again at wavelengths higher than 400 nm due to the degradation process of AgNWs; that is, the 240 min time is not unfavorable for the AgNWs’ growth. As a result, there is a strong correlation between the growth time and growth temperature. To ascertain such a correlation, the AgNWs syntheses were repeated at 150, 160, 170, and 180 °C (Figure 2 and Figure 3). At each growth temperature, the optical absorbance of a small amount of solution (approximately 0.3 mL was diluted 10 times with water) was measured. At all temperatures, the absorption spectra revealed almost the same pattern irrespective of the growth time durations. The growth rate of the AgNWs was found to increase with the increase in the temperatures, indicating that there was a faster growth at higher temperatures. The sharpest peaks at the growth temperatures of 150, 160, 170, and 180 °C corresponded to the growth times of 120, 75, 49, and 34 min.

### 3.3. Reaction Time-Temperature Formula

**Figure 4:**
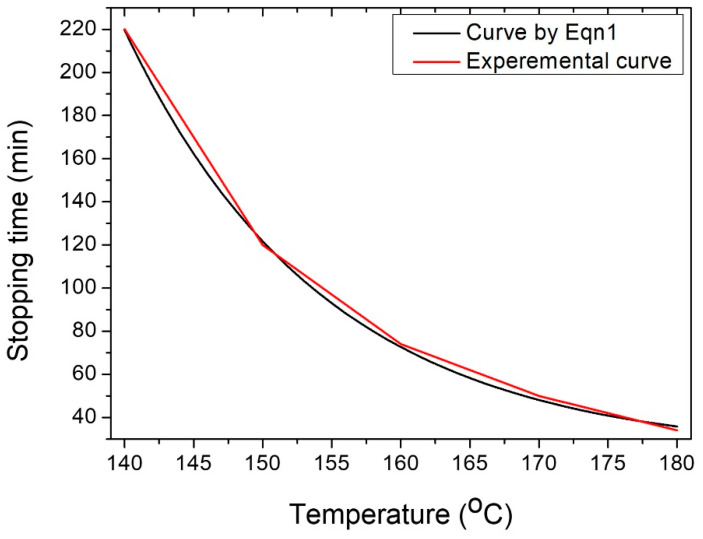
Experimental growth time against growth temperature of AgNWs in the suspension (red) fitted to the theory (black).

Figure 4 displays the measured growth time against the growth temperature of AgNWs in the suspension (red) fitted to Equation 1 (black). The obtained perfect match between the experimental and theoretical curves clearly indicated the exponential decay of the growth time (*t*(*T*)) with the temperature (*T*), given by:

(1) $$t\left(T\right)=A+Be^{-CT}$$

where the fitting parameters are *A* = 23.41 min, *B* = 3087026 min, and *C* = 1/14.49 = 0.069/°C. This equation enabled us to predict accurately the growth time at an arbitrary temperature (for example, a growth time of 93 min at 155 °C).

### 3.4. A Novel Purification Technique of AgNWs

**Figure 5:**
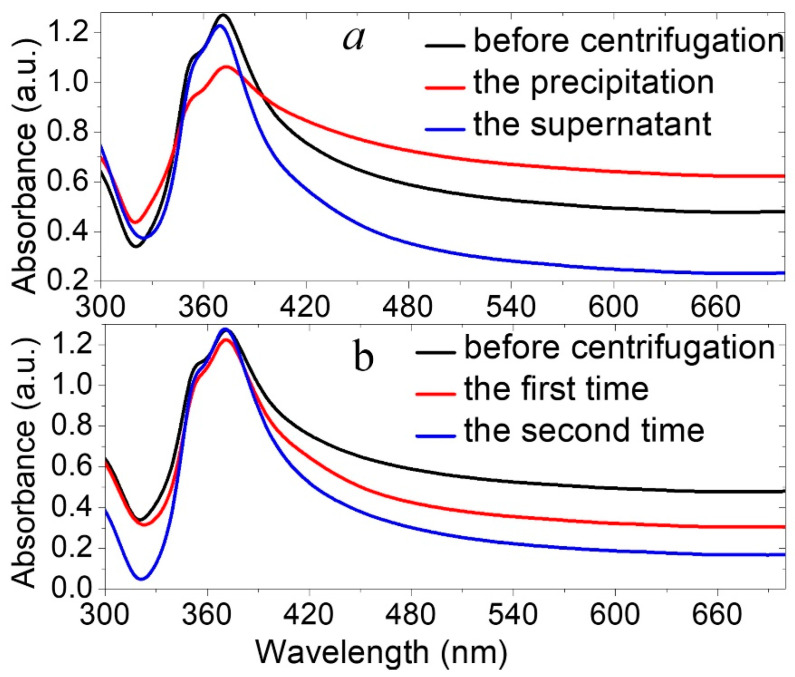
The absorption spectra of AgNWs (grown at 150 °C) during the purification process: (**a**) before the centrifuge, and the supernatant and precipitation after the centrifuge at 1500 rpm. (**b**) before the centrifuge, and centrifugation of supernatant twice at 7000 rpm.

As aforementioned, in this study, the AgNWs were purified using a novel approach. The optimum suspension that was prepared at a reaction temperature of 150 °C was first diluted by deionized water (DIW) (at a ratio of 1:1) and then centrifuged at 1500 rpm for 10 min, enabling the precipitation of heavier AgNRs, AgNPs, nanocubes, and AgNWs. Thus, the upper part of the suspension (supernatant) enclosed the lighter and thinner pure AgNWs. The supernatant was centrifuged using acetone and ethanol separately at a high speed of 7000 rpm twice. The final product was dispersed in 10 mL of ethanol for a further characterization. Figure 5a illustrates the absorption spectra of the as-prepared suspension before the centrifugation (black curve), the separated precipitates (red curve), and the purified supernatant (blue curve) after the centrifugation of the original solution once at 1500 rpm. The peak absorbance of the supernatant after the low-speed centrifugation was much sharper than the peak absorbance of the original solution without centrifugation, which means that the byproducts were filtered out of the original solution. Conversely, the peak absorbance of the precipitates was wider, accompanied by a blue-shift to 373 nm, in addition to broadening at a wavelength range >400 nm, which indicated the existence of heavy AgNPs and AgNWs in the precipitates, as was expected. The peak absorbance of the doubly centrifuged (at 7000 rpm) supernatant was much sharper (blue curve) than the one obtained the first time (red curve), indicating the complete removal of by-products (Figure 5b).

### 3.5. Electron Microscopic Characterization

**Figure 6:**
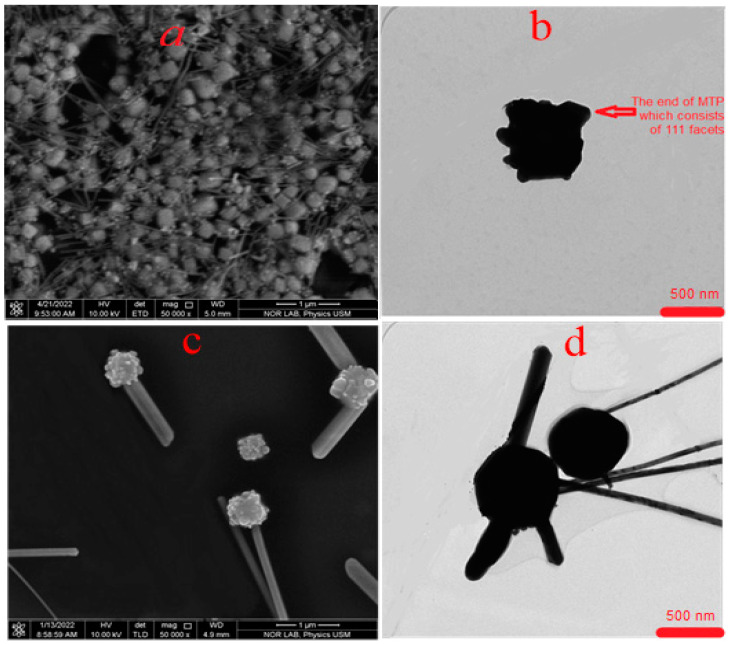
(**a**,**c**) are FESEM images of AgNPs and AgNRs suspensions grown at 140 °C for 125 min and 175 min, respectively, and the corresponding (**b**,**d**) are their TEM images.

**Figure 7:**
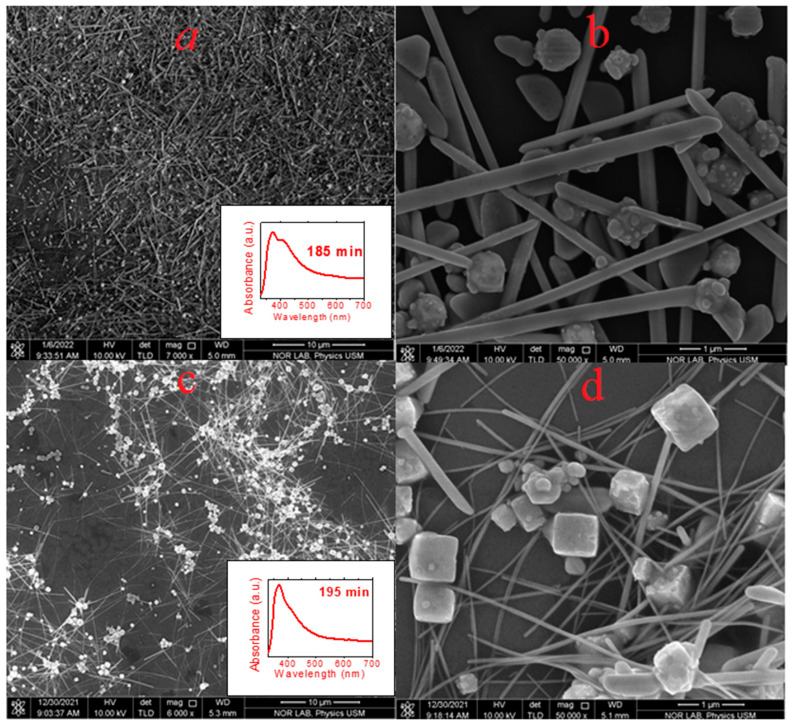
(**a**,**c**) are FESEM images of AgNPs, AgNRs, and AgNWs suspensions grown at 140 °C for 185 min and 195 min, respectively, and the corresponding (**b**,**d**) are their FESEM images with a magnification of 50kx.

Figure 6 and Figure 7 show the FESEM and TEM micrographs of the AgNWs’ suspension produced various times during the synthesis at a temperature of 140 °C. Figure 6a,b depicts the FESEM and TEM images of the suspension at the start of the growth at a time of 125 min after the commencement of the injection of AgNO_3_ as indicated in the optical absorption results. The FESEM images revealed the nucleation of AgNPs- and MTPs-decorated nanocrystals in the suspension. The TEM image (Figure 6b) clearly showed one of these decorated cubical nanocrystals with one end of the MTP enclosing five [111] facets that enabled the transformation into AgNRs, and this was supported by the UV–Visible spectrum as an inset image in Figure 6b. The corresponding FESEM and TEM images (Figure 6c,d) at 175 min showed the growth of MTPs into the AgNRs on the crystals’ surface. These results supported the UV–Vis absorption data of the AgNWs’ suspension grown at 175 min as shown in the inset image in Figure 6d. Figure 7a,b shows the FESEM and TEM images of the mixture of the AgNPs and AgNRs suspension grown at 185 min. Both images consisted of the mixture of Ag-nanocrystals, and the AgNRs grown at 185 min, supporting the UV–Vis results. Figure 7c,d displays the FESEM and TEM images of the AgNPs, AgNRs, and AgNWs’ suspension grown at 195 min. The FESEM and TEM images of the suspension grown at 195 min revealed the dominance of AgNWs over Ag nanocrystals, which matches with the absorption spectral data.

**Figure 8:**
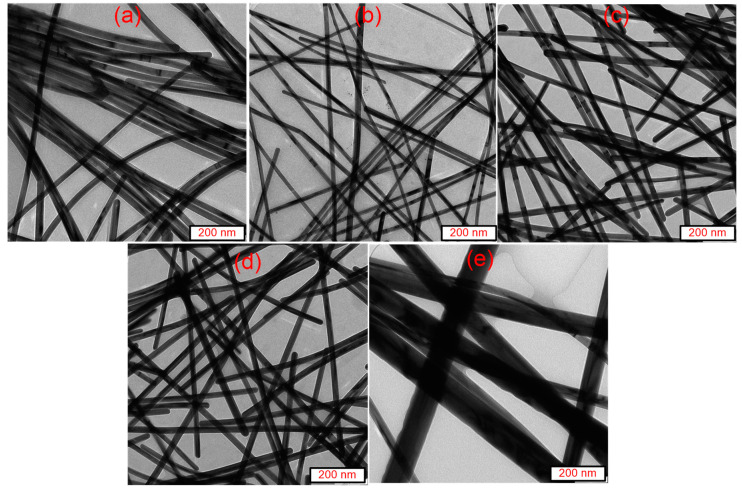
TEM images of AgNWs suspension at the growth time for growth temperature: (**a**) 140, (**b**) 150, (**c**) 160, (**d**) 170, and (**e**) 180 °C.

Figure 8 depicts the TEM images of the purified AgNWs’ suspension for the five temperatures, 140 to 180 °C, at the growth time of each. It is observed that the average diameter of the AgNWs depends mainly on the reaction temperature. The mean diameters of the AgNWs at temperatures of 140, 150, 160, 170, and 180 °C were discerned to be 26, 23, 32, 36, and 53 nm. The observed increase in the mean diameters of the NWs with the increase in the temperature can be ascribed to the quick reduction of Ag+ to Ag in the presence of EG. Consequently, the deposition process of Ag was increased, yielding thicker AgNWs. In addition, the melting point of PVP (150 °C) also played a significant role in the achievement of thicker NWs. At higher growth temperatures (170 and 180 °C), the capping layer (PVP) melts easily and enables the deposit of more Ag atoms on the [100] facets, producing thicker AgNWs. In contrast, lower growth temperatures (140–150 °C) led to the formation of thinner AgNWs. In brief, the results suggested that lower temperatures (140 to 160 °C) are preferable for the production of pure AgNWs than higher temperatures. Based on this disclosure, the growth temperature of 150 °C was chosen to be the optimum to achieve the minimum NWs diameter (23 nm).

### 3.6. X-ray Diffraction Characterization

**Figure 9:**
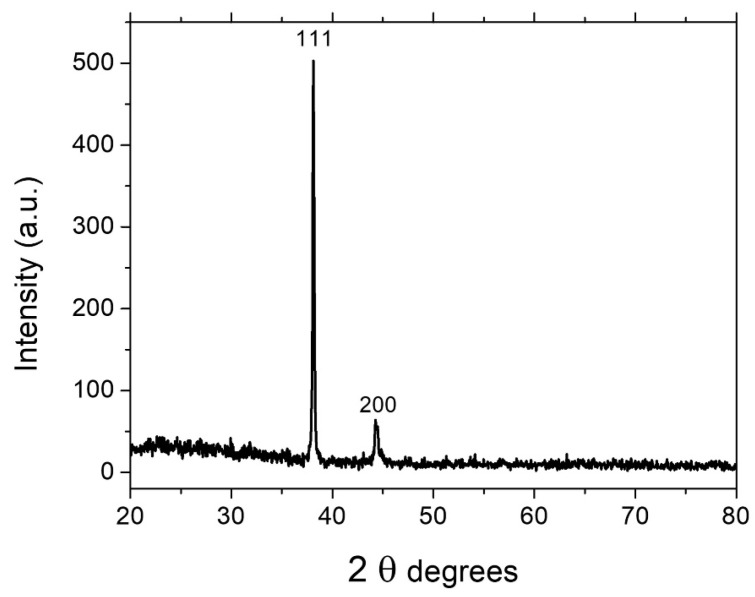
XRD pattern of the AgNWs.

Figure 9 illustrates an XRD spectra with two peaks at 2 θ = 38.13 and 2 θ = 44.36, which correspond to the (111) and (200) planes, respectively. Moreover, these two planes are consistent with silver crystal (FCC), as reported in (JCPDS Card No. 04-0783). The XRD spectra demonstrates that the AgNWs produced by the polyol method consist of a pure crystalline phase. The intensity ratio between the (111) and (200) planes of these two peaks is too high (503:44.73), which shows that the AgNWs grow significantly along (111) planes, while the growth along (200) planes is severely limited by PVP capping [30].

### 3.7. Practical Implementation of AgNWs/P-Si as a Photodetector

**Figure 10:**
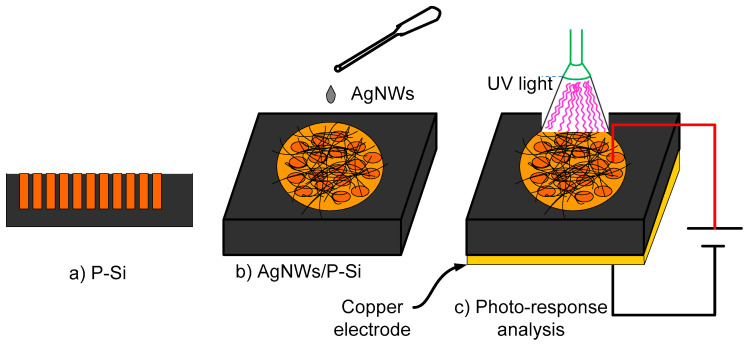
Schematic diagram of (**a**) the fabricated P-Si photodetector, (**b**) the AgNWs/P-Si photodetector, and (**c**) the photo-response analysis.

Figure 10 illustrates the fabricated P-Si photodetector based on AgNWs. On a P-Si surface, a dropper was used to form a thin, circular AgNWs film with a low sheet resistance and a high transmittance. The film was 0.5 cm in diameter. The photo-current-time (I-t) analysis and current-voltage (I-V) curves of the AgNWs/P-Si device due to four different bias voltages were collected using a Keithely supply (2400) linked to software, as shown in Figure 10.

**Figure 11:**
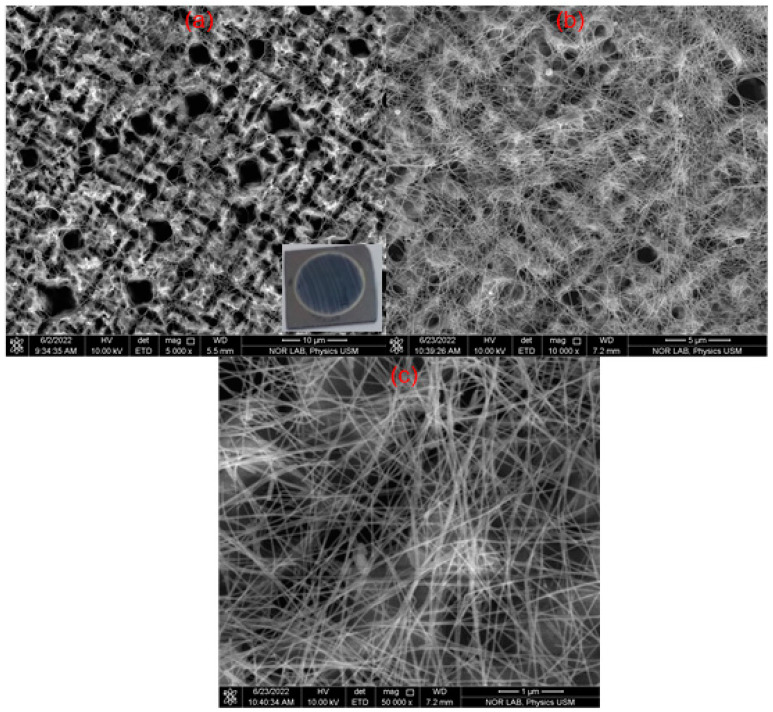
FESEM images for AgNWs over. the P-Si with three different magnifications of (**a**) 5 kx, (**b**) 10 kx, and (**c**) 50 kx.

In this work, the synthesized AgNWs were used to enhance the detectivity of the porous Si to the UV irradiation at a wavelength of 375 nm with different bias voltages of 1 V, 2 V, 3 V, and 4 V. Due to the high transparency, high conductivity, and plasmonic properties of the AgNWs, the photo response of the porous Si (P-Si) photodetector increased dramatically from the μA range to the mA range. A porous silicon (P-Si) structure was formed on the Si (n-type) by electrochemical etching. The FESEM images of the AgNWs/P-Si are displayed in Figure 11a with three different magnifications of 5kx, 10kx, and 50kx. The findings confirm that the etching of the n-type Si by the electrochemical process was successful in producing the porous structure over the Si. As shown in the figure, the pores and the Si are indicated by dark and bright colors, respectively. The formation of a uniform distribution of the PSi structure over the surface of the silicon substrate was confirmed by the observation of many pores with a high density. P-Si, including its large surface-to-volume ratio, high absorbance spectrum, and wide energy gap, in comparison to the bare Si, is a promising and suitable material for a photodetector [31,32,33]. Figure 11 shows FESEM images that investigate the surface morphology of the high-purity AgNWs network that was evenly distributed on the P-Si structure. The high purity of AgNWs is due to the abovementioned efficient purification process.

#### 3.7.1. I-V Characteristics

**Figure 12:**
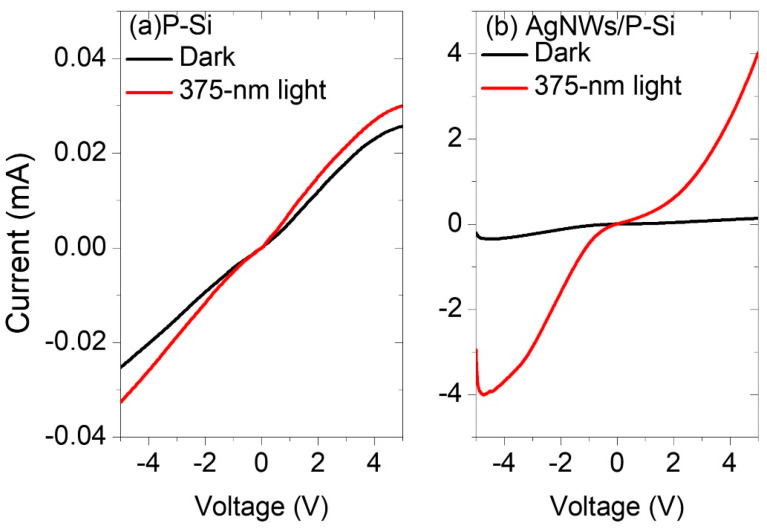
Current-voltage curves of the (**a**) P-Si and (**b**) AgNWs/P-Si photodetector under dark and 375 nm light.

The current-voltage curves of P-Si and AgNWs/P-Si in the voltage interval of [–5, 5] V are shown in Figure 12 under dark and irradiation conditions in the UV range at 375 nm. In the absence of AgNWs, the I-V curves of P-Si were reported to shift slightly when exposed to UV light (Figure 12a). However, the detector responds effectively to UV irradiation once the AgNWs are deposited above the P-Si, and the I-V curve considerably changes from the micro- to milli-scale (Figure 12a). The advantages of P-Si are its large surface area and high absorption compared to bare Si. This improves the AgNWs’ operation by using its surface plasmon resonance and then increasing the detectivity of P-Si to UV light [34,35]. The I-V curves in Figure 12 behave in a Schottky-like manner, confirming that the AgNWs–P-Si contact is of the Schottky type. This is caused by the PSi and AgNWs having distinct work function values. As an external electric field generator, the bias voltage is a crucial factor in separating the charge carriers and generating an internal (built-in) electric field, which improves the photodetector response to UV irradiation at both reverse and forward bias voltages.

#### 3.7.2. Photo-Current Analysis

**Figure 13:**
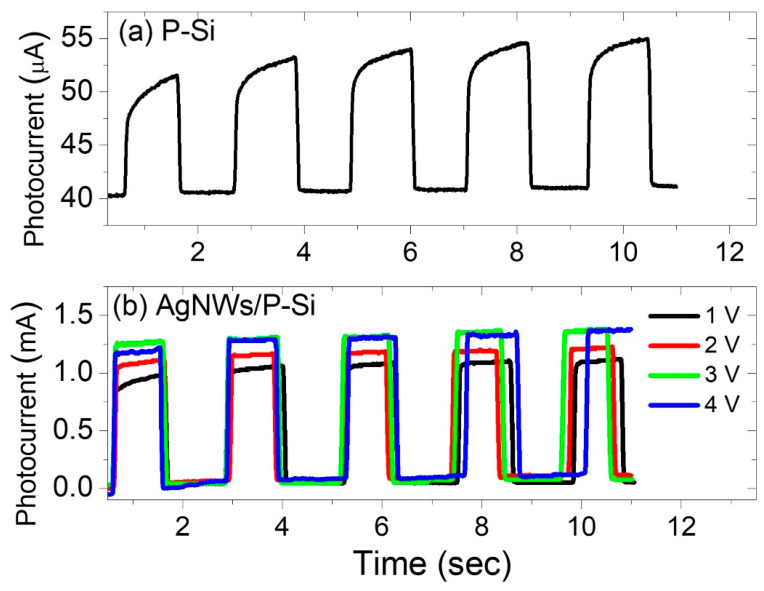
The photocurrent versus time of (**a**) porous Si and (**b**) AgNWs/porous Si photo-sensors for four bias voltages of 1 V, 2 V, 3 V, and 4 V.

**Figure 14:**
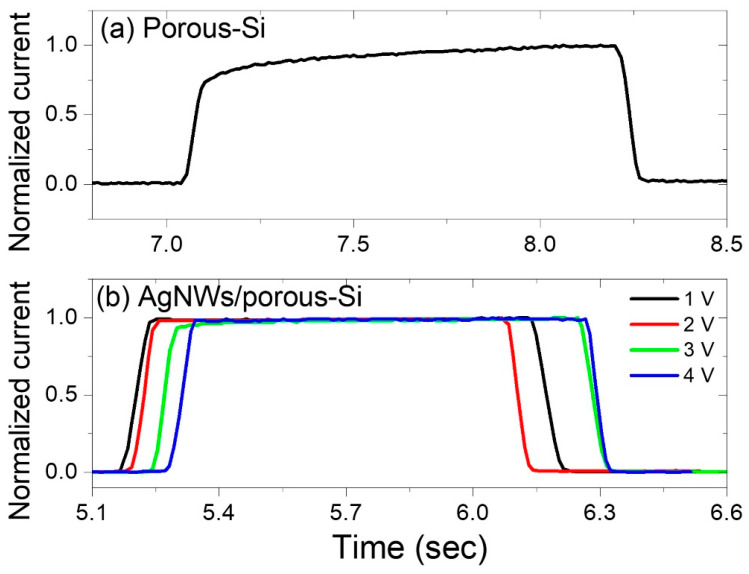
The normalized current versus time of (**a**) the P-Si and (**b**) the AgNWs/P-Si photodetector due to 385 nm and 395 nm wavelengths at a 1 V voltage.

Figure 13a depicts the photo-response of the P-Si to 375 nm UV light. The photodetector performed weakly at the 375 nm UV light with a reverse-biased voltage of 4 V, and dark- and photo-currents of 43.26 μA to 54.43 μA. The rise time ($t_{r}$) is the time needed for the photocurrent to increase from 0.1 to 0.9 of its highest value, while the decay time ($t_{d}$) is the time needed for the photocurrent to fall from 0.9 to 0.1 of its highest value. As shown in Figure 14, a normalization was done of a pulse for each photodetector in order to determine the rise and fall times for each. It is observed from Figure 14a that the rise and decay times of the P-Si photodetector are 0.333 s and 0.063 sec, respectively. When a film of AgNWs is coated on a 5 mm diameter circle, a significant response from the 375 nm wavelength is observed at four different bias voltages: 1 V, 2 V, 3 V, and 4 V, and the photocurrent is enhanced dramatically for the four voltages from tens of micro-amperes in the P-Si photodetector to 1.1 mA, 1.2 mA, 1.37 mA, and 1.35 mA in the AgNWs/P-Si photodetector with bias voltages of 1V, 2V, 3 V, and 4 V, respectively (Figure 13a). It is worth mentioning that the dark current of the AgNWs/P-Si photodetector is 93.69 μA. The AgNWs-P-Si photodetector obtained a very short rise and decay time of 0.039 s and 0.041 s, respectively (Figure 13b). This rapid response demonstrates the efficiency of AgNWs in improving the P-Sis sensitivity to UV radiation, especially at the 375 nm wavelength.

#### 3.7.3. Parameters of UV Photodetectors Based on AgNWs

Table 1 reveals the quantum efficiency, the gain, the sensitivity, the responsivity, the detectivity, the rise time, and the fall time versus the AgNWs/porous-Si photodetectors with different bias voltages at a wavelength of 375 nm. The following formula is the responsivity equation (*R*), which is considered to be a crucial parameter in evaluating the performance of the photodetectors:

(2) $$R=\frac{I_{photo}}{A\;P}$$

where *A*, *P*, and $I_{photo}$ are the active area, the power of incident light per 1 m^2^, and the photocurrent. It is observed that at 3 V of the applied bias voltage, the photodetector’s highest responsivity is calculated to be 31.44 A/W. In comparison to what Kumar et al. reported, this result is significant due to the surface plasmon effect of AgNWs on the performance of the porous Si photodetector [36]. Furthermore, the presence of a wide depletion width due to the presence of AgNWs may enhance the responsivity. By measuring the photocurrent, we were able to compute the quantum efficiency (η), which is the number of electron-hole couples produced per incident photon, as follows:

(3) $$\eta =\frac{R\;hc}{\lambda e}$$

where *e, c, λ, h*, and *R* are, respectively, the elementary charge, the speed of light, the wavelength, Planck’s constant, and the responsivity. As shown in Table 1, the quantum efficiency of our photodetector is a significant value at 3 V compared to other voltages. the sensitivity (*S*) and the gain (*G*) are also important parameters used to evaluate the photodetector’s performance, which are defined as:

(4) $$S=\frac{I_{photo}-I_{dark}}{I_{dark}}\times 100\%$$

(5) $$G=\frac{I_{photo}}{I_{dark}}$$

where $I_{dark}$ is the dark current. The results of the sensitivity analysis are presented in Table 1. It is important to note that the sensitivity (2247.49%) and the gain are dramatically higher than that reported in previous studies employing AgNWs-Si nano-pillar photodetectors [26]. The next equation is utilized to calculate the detectivity (D*):

The AgNWs/P-Si obtained a high detectivity of 2.89 × 1012 Jones at a 1 V bias voltage, as shown in Table 1, which is considered to be a good result relative to the other studies [25,26,27,28]. Our research indicates that the UV photodetector performance of AgNWs/porous-Si is substantially better than that of other nanostructures and films thanks to their large surface area relative to their volume, which can absorb a high amount of incident light and generate more free electrons than bare Si.

**Figure 15:**
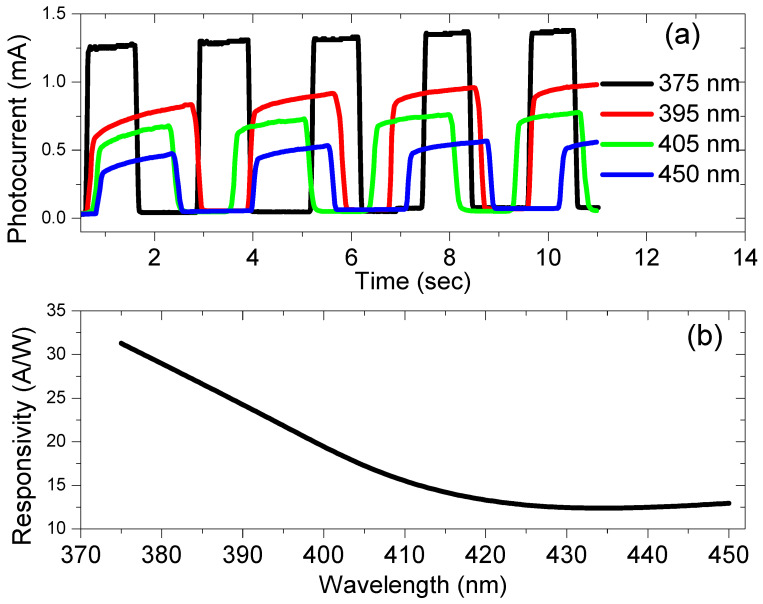
(**a**) The current-time characteristic for four different wavelengths and (**b**) the photo-response of the AgNWs/P-Si photodetector versus wavelength.

Figure 15a shows the photocurrent versus the time of the AgNWs/P-Si photodetector for four different wavelengths: 375 nm, 395 nm, 405 nm, and 450 nm. It is observed that the photocurrent of the AgNWs/P-Si photodetector decreases with the increase in the wavelength, since the photocurrent decreased from 1.37 mA at a wavelength of 375 nm (UV light) to 0.566 mA at a wavelength of 450 nm (blue light). The responsivity of the photodetector due to the four wavelengths was calculated and then plotted versus the wavelength, as shown in Figure 15b. The figure indicates that the responsivity decreases when we go to visible light.

#### 3.7.4. Energy-Band Diagram

**Figure 16:**
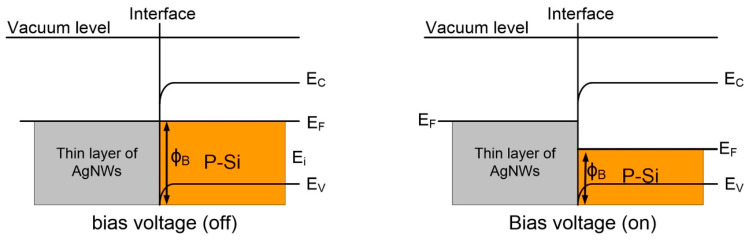
Energy-band diagram of AgNWs/P-Si heterostructure.

In porous silicon, the pores contain extremely thin silicon crystallite walls at the nanoscale. Because the energy band gap is proportional inversely to the length squared, smaller nano-crystallites have a greater energy band gap and better quantum trapping of the electrons. The AgNWs and PSi work functions (4.2 and 4.57 eV, respectively) are used to generate the energy band schematic diagram. When the semiconductor (P-Si) was deposited by a thin film of AgNWs, electrons migrated from the AgNWs thin film to the P-Si, as shown in Figure 16. As a result, the PSis work function decreased and a depletion region of electrons and holes formed close to the PSis surface. Most P-Si structures are a few microns thick, which gives them a higher ratio of surface area to volume than bulk silicon surfaces [36]. Therefore, depositing the AgNWs in this large area will improve the conductivity of the structure due to decreasing the work function of the P-Si. When a bias voltage is applied to the AgNWs/P-Si device, the fermi levels of the AgNWs and P-Si are balanced by the increase in the AgNWs compared to the P-Si. This causes a decrease in the depletion barrier potential, or an electric field. So, the photo charges can move freely from the P-Si to the AgNWs through the barrier. This means that the AgNWs/P-Si photodetectors work with a high performance.

#### 3.7.5. Checking the Transparency of AgNWs over the Glass

The purified AgNWs were used to fabricate the transparent conductive film (TCF) via the spray coating method. The purified AgNWs were sprayed four times on a glass slide (2.5 cm ×2.5 cm) and were cleaned carefully in the sonication device with acetone, ethanol, and DIW each for 10 min. Then, the thermal evaporation device was used to make two electrodes of silver on the glass slide. An Ohmmeter device was used to measure the sheet resistance ($R_{s}$) of the TCF between the electrodes and the UV–Visible–NIR device was used to measure the transmittance spectrum of the AgNWs/glass. Figure S1 shows the transparency of the AgNWs on the glass versus the wavelength with a sheet resistance of 16.1 Ω/sq.

## 4. Conclusions

Using a novel purification scheme, a series of ultra-thin AgNWs were obtained. The structures, morphologies, and UV–Vis absorbance of the produced AgNWs were tailored by adjusting the polyol reaction temperatures and times. The synthesis process was performed at five different temperatures, 140–180 °C. The UV–Vis absorption spectra of the AgNWs with different times at each temperature were comprehensively analyzed and a new growth mechanism was proposed wherein the growth time was determined to reduce the by-products. The growth times and reaction temperatures of the AgNWs were found to be strongly correlated and an equation was generated from the experimental absorbance data to determine the growth time at any temperature other than the five mentioned temperatures. The growth time of the AgNWs decreased exponentially with the temperature. The AgNWs that were synthesized at a temperature of 150 °C had a minimum diameter of 23 nm, thus this is considered to be the optimum. PSi-based UV photodetectors with an exceptionally high sensitivity have been developed using these AgNWs. At a 3 V bias voltage, the constructed AgNWs/P-Si photodetector exhibited a 2.53 × 1012 Jones detectivity, 31.44 A/W responsivity, and 1356.48 sensitivity. In contrast to the P-Si photodetector, which obtained a photocurrent increase from 43.26 μA to 54.43 μA, the photocurrent for the AgNWs/P-Si photodetectors jumped drastically from 93.69 μA to 1.37 mA at a 3 V bias voltage. These findings can be attributed to the exceptional qualities of the purified AgNWs and their surface plasmonic resonance characteristics.

## Figures and Tables

**Table 1:** Key parameters of AgNW-based photosensors in a comparison between the current work and the previous studies.

**No. Layers**	Bias Voltage	η	*G*	Sensitivity	*R*	$D\times 10^{12}$	$t_{r}(S)$	$t_{d}(S)$	λ	Ref.
				(%)	A/W	Jones			nm	
**Porous-Si**	4	4.15	1.32	32.63	1.24	0.152	0.333	0.063	375	Current work
**AgNWs/P-Si**	1	84.62	23.47	2247.49	25.37	2.89	0.053	0.054	375	Current work
**AgNWs/P-Si**	2	93.08	16	1500.22	27.9	2.51	0.039	0.041	375	Current work
**AgNWs/P-Si**	3	104.8	14.56	1356.48	31.44	2.53	0.043	0.048	375	Current work
**AgNWs/P-Si**	4	102.9	13.78	1278.32	30.86	2.44	0.042	0.044	375	Current work
**AgNWs-Si nano-pillars**				108	2.32	0.0011	1.4	-		[25]
**AgNWs-ZnO-Si**		-	-	-	5.52	0.234	0.362 m	0.403 m	370	[26]
**AgNW-Si nano-holes**		-	2.1 × 10^−4^	-	30 × 10^−3^	0.2	0.244	0.918	365	[27]
**AgNWs-ZnO-AgNWs**		-	-	6.34 × 10^4^	9.4 × 10^−3^	0.169	<1	<1	365	[28]

## Supplementary Materials

The following supporting information can be downloaded at: https://www.mdpi.com/article/10.3390/nano13020353/s1, Figure S1: XRD pattern of the AgNWs; Figure S2: The transmittance of AgNWs thin film coated on the glass with sheet resistance of 16.2 Ω/sq.
