# Supplementary material for: Synthesis and Deposition of Silver Nanowires on Porous Silicon as an Ultraviolet Light Photodetector
Source: Nanomaterials (Basel). 2023 Jan 15;13(2):353. doi: 10.3390/nano13020353 (PMC9863988; doi:10.3390/nano13020353)
Supplement: Supplementary file 1 [file nanomaterials-13-00353-s001.zip › nanomaterials-2138667-supplementary.pdf]

Synthesis and Deposition of Silver Nanowires on Porous Silicon as an Ultraviolet Light Photodetector

Anas A. M. Alqanoo[1,2], Naser M. Ahmed[1,3]*, M.R. Hashim[1], Munirah A. Almessiere[4,5], Sofyan A. Taya[2], Osamah A. Aldaghri[6] and Khalid Hassan Ibnaouf[6]*

[1] Schoo of Physics, Universiti Sains Malaysia, 11800 Penang, Malaysia
[2] Physics Department, Islamic University of Gaza, P.O. Box 108, Gaza, Palestine
[3] The University of Mashreq, Research Center, Baghdad, Iraq

[4]Department of physics, college of science, imam abdulrahman bin faisal university, P.O BOX 1982, Dammam 31441, Saudi Arabia

[5]Department of Biophysics, Institute for Research & Medical Consultations (IRMC), Imam Abdulrahman Bin Faisal University, P.O. Box 1982, 31441, Dammam, Saudi Arabia
[6]Department of 5Physics Department. College of Science, Imam Mohammed Ibn Saud Islamic University (IMSIU), Riyadh 13318, Saudi Arabia

S1. X-ray diffraction analysis

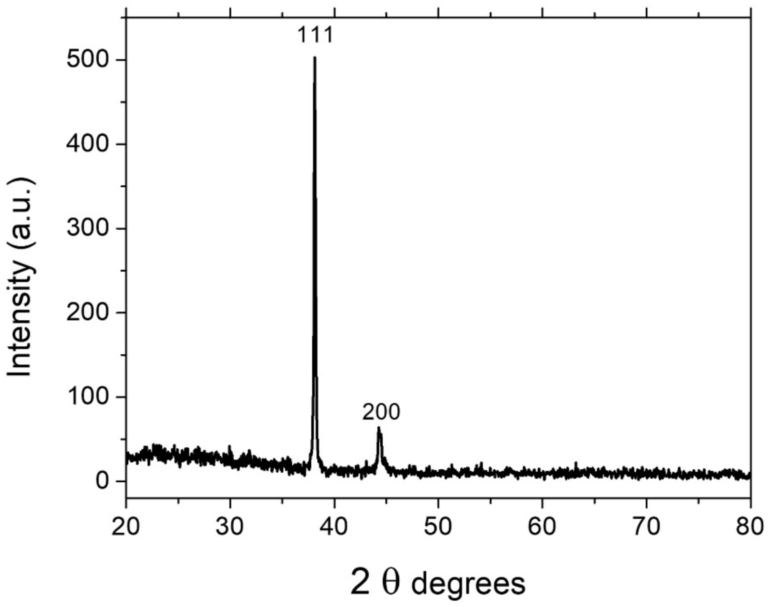

Figure S1. XRD pattern of the AgNWs.

S2. The transmittance spectrum of the AgNWs film on glass

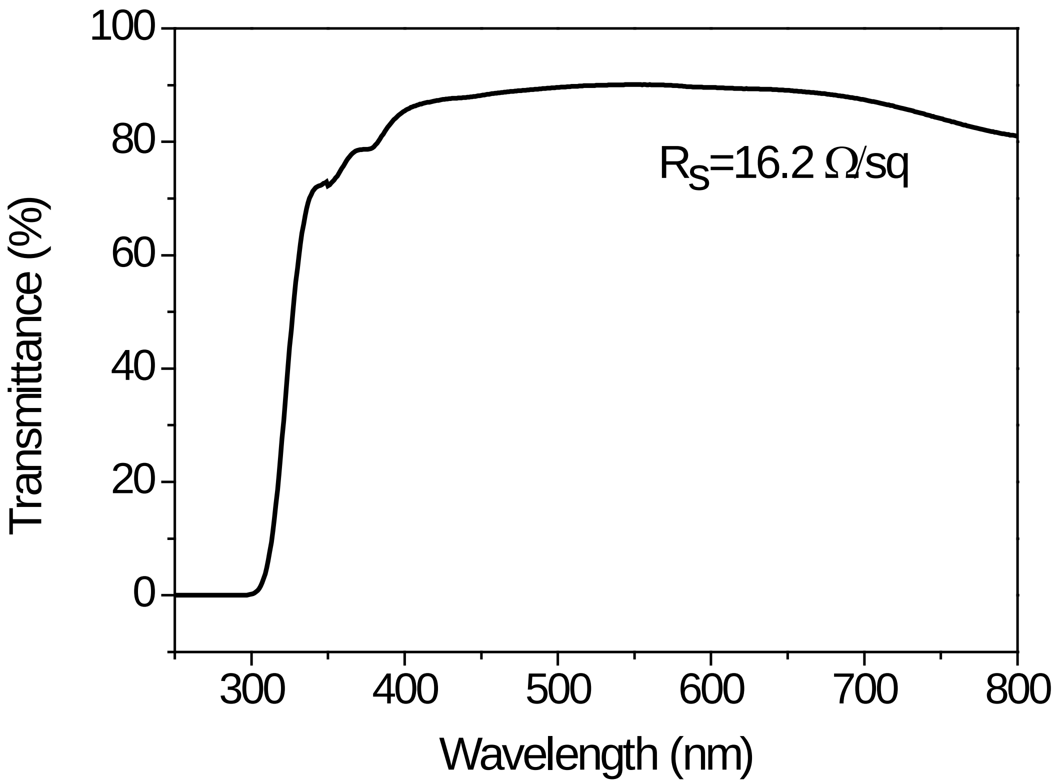

Figure S2. The transmittance of AgNWs thin film coated on the glass with sheet resistance of 16.2 Ω/sq.
